# Primary primitive neuroectodermal tumor of the cervix confirmed with molecular analysis in a pregnant woman: A case report and literature review

**DOI:** 10.3389/fgene.2022.871531

**Published:** 2022-08-10

**Authors:** Ding Wei, Zhao Jianguo, Li Xiao, Qu Pengpeng

**Affiliations:** ^1^ Department of Gynecological Oncology, Tianjin Central Hospital of Gynecology and Obstetrics, Tianjin, China; ^2^ Clinical School of Obstetrics and Gynecology Center, Tianjin Medical University, Tianjin, China

**Keywords:** primary primitive neuroectodermal tumor, cervix, pregnant woman, molecular analysis, literature review

## Abstract

Primary primitive neuroectodermal tumor (PNET) in the female tract is rare. Recently, a case of cervical PNET was diagnosed in our hospital. A 29-year-old pregnant woman presented with a cystic-solid cervical mass at the 7th week of gestation. The mass grew rapidly during follow-up and ruptured at the 22nd week. A biopsy was performed on the mass. Pathological examination revealed a malignant neoplasm composed of small cells which exhibited positive immunohistochemical (IHC) staining for CD99, SYN, and FLI1. Fluorescence *in situ* hybridization (FISH) displayed the presence of *EWS-FLI1* fusion gene resulting from the chromosomal translocation t (11;22, q24;q12), which confirmed the diagnosis of cervical PNET. The reverse transcription-polymerase chain reaction (RT-PCR) results showed type 2 *EWS-FLI1* fusion occurred in this tumor, suggesting a poor prognosis. The patient underwent surgical resection and was given adjuvant chemotherapy followed by pelvic radiotherapy. PNET arising from the genital tract, especially in the uterine cervix, is very rare and presents a diagnostic challenge. FISH and RT-PCR analysis are helpful for the diagnosis of such a tumor at an unusual site, as in the present case.

## Introduction

Primary primitive neuroectodermal tumor (PNET) is a highly malignant tumor characterized by neuroectodermal and neural crest cells’ origin, most of which arise from the central nervous system, soft tissues, and bones (Rajwanshi et al., 2009). In more than 90% of PNET cases, chromosomal translocations result in the fusion between the *EWS* gene (also known as *EWSR1*, Ewing sarcoma breakpoint region 1) and a member of the ETS family of transcription factors, such as *FLI1* and *ERG* (Song et al., 2012).

In this article, we presented a case of cervical PNET diagnosed during pregnancy whose tumor grew very rapidly. The patient terminated her pregnancy, and was treated with surgical resection, adjuvant chemotherapy, followed by pelvic radiotherapy. To gain a deeper understanding of this rare disease, we reviewed PNET-related literature and found 26 cases of primary cervical PNET, including 5 cases occurring during pregnancy.

## Case report

A 29-year-old female, gravida 1 para 0, was referred to her obstetrician at the 7th week of gestation. An ultrasonography examination revealed a cystic-solid mass measuring 5 cm × 4 cm in the cervix. Her medical and family history were not significant. No additional examinations or treatments were performed because of her pregnancy. During follow-up, the ultrasonography examination demonstrated that the mass grew rapidly, and at the 20th week, the patient was admitted to our department because she began to complain of low back pain. An MRI was performed and it was found that the mass grew to 11 cm × 10 cm × 10 cm in just 2 months. The levels of CA-125, CEA, SCC, and NSE were normal. Considering the pregnancy, the patient refused biopsy and chose to continue observation.

**FIGURE 1:**
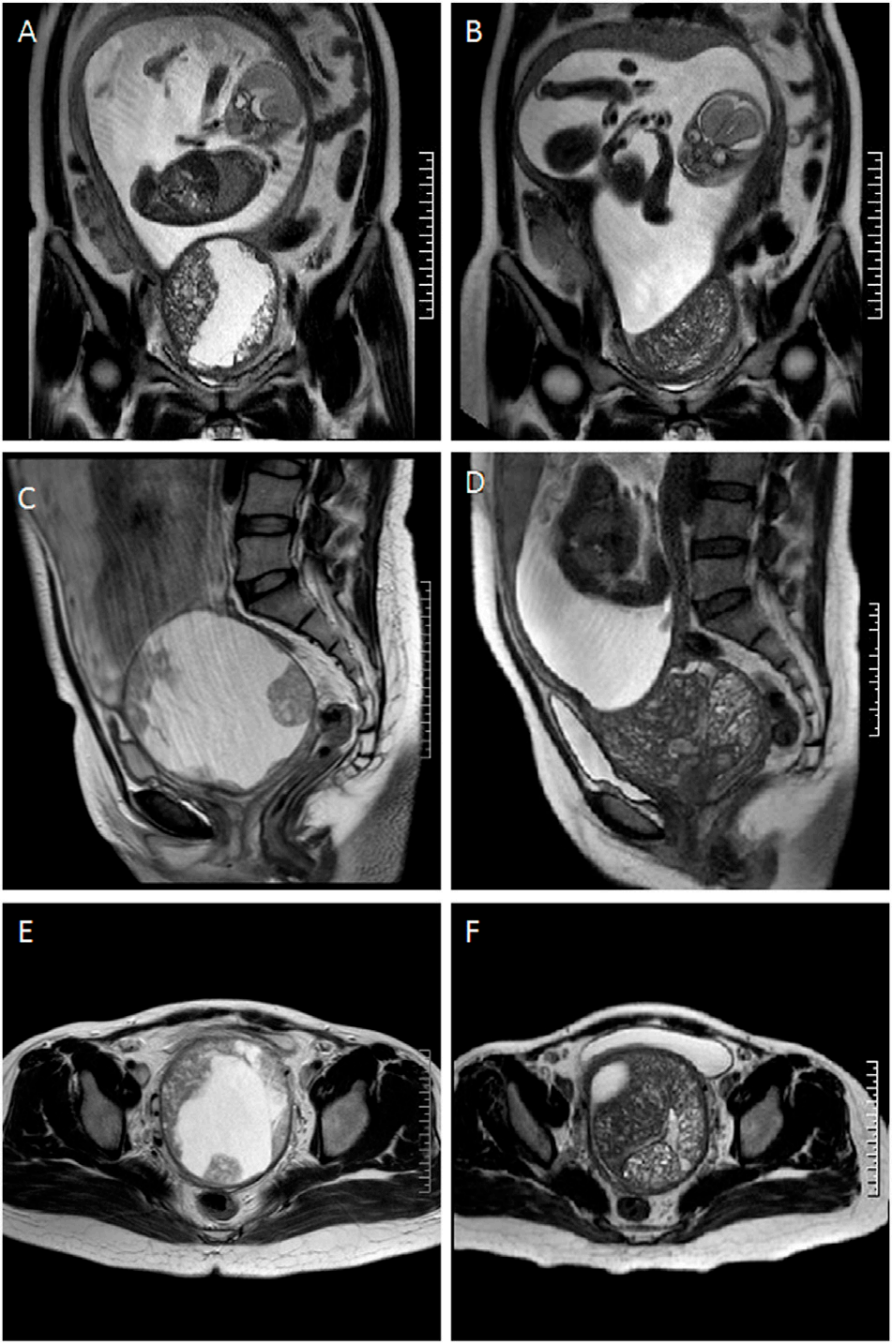
Comparison of the magnetic resonance images of the tumor at the coronal, sagittal, and axial plane before and after rupture. **(A)**, **(C)** and **(E)** tumor at 20 weeks (before rupture) was shown. **(B)**, **(D)** and **(F)** tumor at 22 weeks (after rupture) was shown.

**FIGURE 2:**
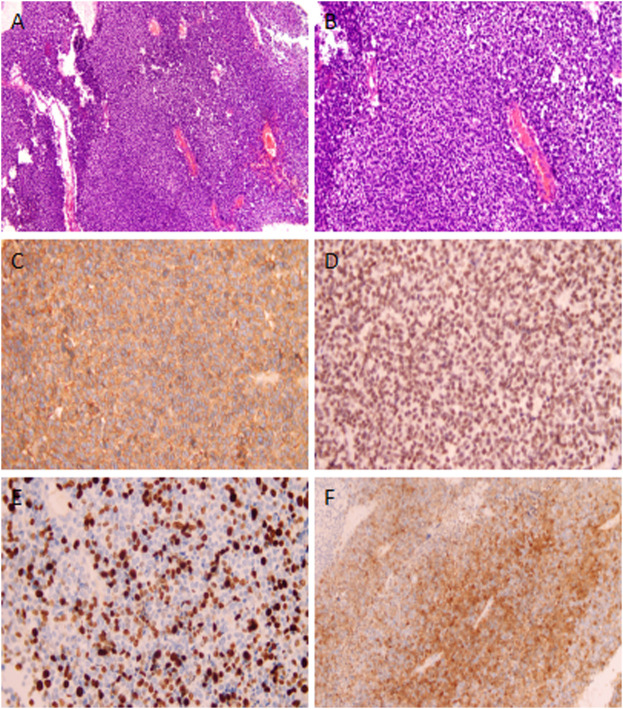
**(A,B)** Microscopic view of the tumor of the cervix with H&E staining, revealing that tumors were composed of a monotonous population of small round cells. IHC staining was positive for **(C)** CD99, **(D)** FLI-1, **(E)** Ki-67, and **(F)** p16. Magnification details: **(A)** ×100; **(B)**200×; **(C)** 200×; **(D)** 400×; **(E)** 200×; and **(F)** 200×.

**FIGURE 3:**
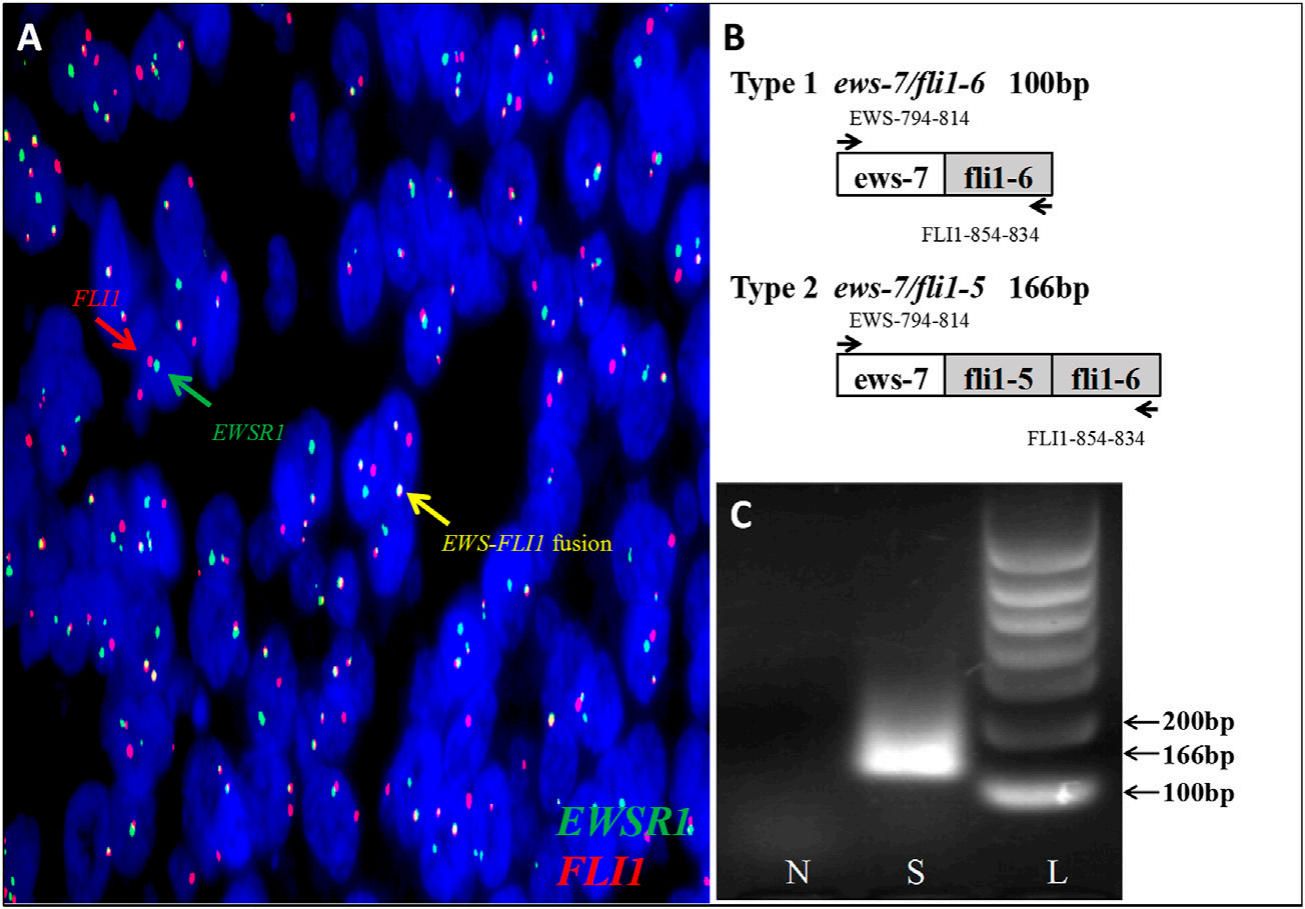
**(A)** FISH testing demonstrated that more than 35% of cells were positive indicating *EWS-FLI1* gene rearrangement (*FLI1* gene, red; *EWSR1* gene, green; and *EWS-FLI1* fusion gene, yellow). **(B)**Fusion of *EWS* -exon 7 to *FLI1*-exon 6 (*ews-7/fli1-6*) creates a PCR product of 100 bp, while fusion of *EWS* -exon 7 to *FLI1*-exon 5 (*ews-7/fli1-5*) creates a PCR product of 166 bp. **(C)**Investigation of the *EWS-FLI1* fusion transcripts in this patient. The primers (EWS- and FLI1-) were used in subsequent PCR reactions of RT-PCR assays performed on RNA isolated from paraffin-embedded tumor tissue to search for fusion transcripts with different exon combinations (*EWS*-exon 7 fused to either *FLI1*-exon 5 or -exon 6). The patient’s sample was found positive for a *EWS-FLI1* product at the 166bp. S: sample; N: normal cervix tissue; L: 100bp DNA ladder.

However, at the 22nd week of gestation, the patient was referred to the emergency department because of vaginal watery discharge. Gynecological examination showed that there was a rupture on the surface of the tumor and fluid was flowing out of the rupture, which was thicker than amniotic fluid. The result of amniotic fluid crystallization was negative. In addition, MRI showed the cystic-solid mass was smaller (10 cm × 8 cm × 7 cm) than before. Bimanual pelvic examination revealed the tumor was in the cervix, without invading the vagina, or adjacent organs (Figure 1). The patient underwent a biopsy of the cervical mass, and it was found that the tumor consisted of small round cells and had extensive necrosis. IHC showed that tumor cells expressed CD99, synaptophysin (SYN), friend leukemia integration 1 (FLI1), and P16. However, no expression of cytokeratin (CAM5.2), P53, chromogranin A (CgA), CD56, or neuron specific enolase (NSE) was detected. Ki-67 proliferation index was about 80% (Figure 2). A pathological diagnosis of cervical PNET was suspected. Next, we performed FISH to detect the presence of the chromosomal translocation t (11;22, q24;q12). A section of 4 μm was cut from paraffin-embedded biopsy blocks and stained with hematoxylin-eosin in order to confirm the presence of tumor cells and to choose the appropriate area for the hybridization procedures. A specific translocation separation probe kit (Vysis, United States ) was used, which consisted of two probes; one directly labeled with red fluorescence and bound to 3′ end of the *FLI1* (11q24) gene, and the second directly labeled with green fluorescence and bound to 5′ end of the *EWSR1* (22q12) gene. According to the manufacturer’s instructions, 200 nuclei with fluorescence signal were randomly selected to observe the number of nuclei with hybridization signal by using a Zeiss Axioplan fluorescence microscope (Zeiss, Germany). Nuclei with the *EWS-FLI1* fusion gene showed yellow signals, while nuclei without gene translocation showed separate red and green signal. The criterion for a positive result of the *EWS-FLI1* fusion gene was that more than 10% of the nuclei showed a fusion signal. FISH demonstrated that more than 35% of the cells were positive, indicating the presence of the *EWS-FLI1* fusion gene (Figure 3A). In order to clarify the type of fusion, we performed RT-PCR test. Total RNA was isolated using TRIzol (Gibico, United States ) and the concentrations were measured by NanoDrop-2000c (Thermo Fisher Scientific, United States ). Transcriptor high fidelity cDNA synthesis kit (Takara, Japan) was used for reverse transcription of 1 μg of RNA from the sample. RT-PCR was carried out using SYBR Green Master Mix (Roche, Switzerland) in a Quantstudio3 real-time thermal cycler (Thermo Fisher Scientific, United States ). Expression data were normalized to GAPDH. The primer sequences used for RT-PCR analysis of *EWS-FLI1* were as follows; 5′- CCA​AGT​CAA​TAT​AGC​CAA​CAG-3′ and 5′-GGC​CAG​AAT​TCA​TGT​TAT​TGC-3’. RT-PCR products were analyzed on a 2% agarose gel electrophoresis. The results showed the RT-PCR product of the tumor was 166bp, which indicated that the 7th exon of *EWS* was fused to the 5th exon of *FLI1* (Figures 3B,C). These results confirmed the diagnosis of cervical PNET.

Chest computed tomography, abdominal and pelvic MRI did not reveal lymph nodes or distant metastases. The patient and her family were informed of the highly invasive nature and poor prognosis of the disease, and they decided to terminate the pregnancy and receive surgical treatment. Subsequently, the patient underwent a type C1 radical hysterectomy, bilateral salpingo-oophorectomy, and pelvic lymphadenectomy. There was a complete fetus in the uterine cavity, and the cervical tumor was 9 cm × 8 cm × 8 cm in stereoscopic size, invading more than two-thirds of the cervical interstitium and extending to the parametrium. No lymph node metastasis was found. However, there was lymphovascular invasion. Histological and IHC staining supported the previous diagnosis of PNET, and according to the International Federation of Gynecology and Obstetrics, the clinical stage was determined asⅡB. After surgery, the patient received adjuvant chemotherapy with pirarubicin, ifosfamide, and cisplatin for six cycles, followed by pelvic radiotherapy of 50 Gy. She remained free of disease for thirty-one months after the diagnosis.

## Discussion

PNET belongs to a group of small round cell tumors that are most commonly found in the central nervous system. In the female reproductive tracts, the ovaries are the most common sites (De Nola et al., 2018). To date, only 26 cases of cervical PNET have been reported in the English literature (Table 1). The age of reported patients ranged from 19 to 59 years old, with a median age of 34. The main symptoms of cervical PNET include irregular vaginal bleeding, lower abdominal pain, uterine enlargement, and increasing mass size. Cervical PNET in pregnant women is even rare, with only five cases reported. Tsao et al. is credited with the first description of cervical PNET in a pregnant woman (Tsao et al., 2001). The authors described a 24-year-old woman in her 8th week of pregnancy presented with a cervical mass about 8 cm × 7 cm and underwent tissue biopsy with further diagnosis of PNET. A radical hysterectomy with bilateral ovarian transposition and periaortic lymphadenectomy was performed, followed by chemotherapy and radiotherapy. The patient remained disease free for 24 months after the treatment. Two cases were described by Kyriazoglou et al. and Khosla et al. (Khosla et al., 2014; Kyriazoglou et al., 2019). In both instances, the patients (9 weeks of pregnancy and 10 weeks of pregnancy, respectively) presented with a cervical mass of about 8 cm × 7.6 cm and measuring 5 cm in the greatest dimension, respectively. Both patients underwent radical hysterectomy and received adjuvant chemotherapy and radiotherapy. A follow-up of 42 and 33 months, respectively, showed both patients to be alive after initial diagnosis. In addition to the reports of cervical PNET in pregnancy, Feng et al. and Al-Nueimy et al. also described one case each, found at 14 weeks of pregnancy and during cesarean section at term, respectively (Al-Nueimy, 2014; Feng et al., 2021). Both patients presented with cervical masses of 3 cm × 3 cm and 6 cm × 4.5 cm, respectively. A radical hysterectomy with bilateral adnexectomy was performed, without lymphadenectomy. Follow-up information obtained only in the Feng et al. report, showed the patient to be alive 36 months after her diagnosis. Our case was the largest PNET ever reported, and we observed that the tumor grew rapidly during pregnancy and even spontaneous rupture occurred. Some reports show that PNET enlargement is more obvious during pregnancy (Torga et al., 2015; Miao et al., 2018). However, the association between the change of hormones during pregnancy and tumor growth needs further confirmation.

**TABLE 1:** Summary of clinical presentation, management, and outcome of PNET of the uterine cervix.

Author		Age, years	Pregnant	FIGO stage	Clinical presentation	Treatment	Follow-up, mo
1	Sato et al. (1996)	44	No	ⅠB2	Vaginal bleeding	TAH + BSO + PL + CT	AWD, 6
2	Horn et al. (1997)	26	No	ⅠB1	Suspect cervical smears	TAH + BSO + PL + RT + CT	DOD, 50
3	Cenacchi et al. (1998)	36	No	ⅠB2	Intermenstrual spotting	TAH	AWD, 18
4	Pauwels et al. (2000)	45	No	ⅠB1	Irregular uterine bleeding	TAH + RT	AWD, 42
5	Tsao et al. (2001)	24	Yes	NA	Vaginal bleeding, vaginal pressure, and increased urinary frequency	CT + RH + CT + RT	AWD, 24
6	Malpica and Moran, (2002)	35	No	ⅠB1	Abnormal uterine bleeding	TAH + BSO + PL + CT	AWD, 5
7	Malpica and Moran, (2002)	51	No	ⅠB2	Abnormal uterine bleeding	TAH + BSO + PL + CT	AWD, 18
8	Snijders-Keilholz et al. (2005)	21	No	ⅠB2	Intermenstrual vaginal bleeding	CT + TAH + CT	AWD, 27
9	Goda J (2006)	19	No	NA	Watery and foul smelling discharge per vaginum and lower abdomen pain	CT + RT	AWD, NA
10	Farzaneh et al. (2011)	45	No	ⅠB2	Yellow purulent vaginal discharge	CT + RH + PL + CT	AWD, 48
11	Benbrahim et al. (2013)	25	No	ⅡB	Irregular vaginal bleeding	Conization with brachytherapy	AWD, 96
12	Arora et al. (2012)	23	No	NA	Irregular vaginal bleeding and dysuria	CT + RH + BSO + PL + CT	AWD, 48
13	Masoura et al. (2012)	23	No	ⅣB	Irregular uterine bleeding and lower abdominal pain	TAH + BSO + CT	DOD
14	Li et al. (2013)	27	No	ШB	Contact uterine bleeding, yellow vaginal discharge, and lower abdominal pain	CT + RT	AWD, 6
15	Khosla et al. (2014)	28	Yes	ⅠB2	Occasional vaginal bleeding and abdominal pain	RH + BSO + PL + CT	AWD, 33
16	Xiao et al. (2014)	52	No	ⅡA	Vaginal bleeding and uterine enlargement	TAH + BSO + PL + CT + CRS	Pelvic recurrence, 6; DOD, 9
17	Xiao et al. (2014)	59	No	ⅣB	Pelvic mass prolapsed from the vagina and vaginal bleeding	TAH + BSO + PL + partial small intestine excision	DOD
18	Al-Nueimy (2014)	27	Yes	ⅠB	Obstructed labor	Caesarean section followed by TAH, BSO, and LNS	NED, FU not reported
19	Mashriqi et al. (2015)	49	No	ⅡB	Vaginal bleeding	CT + TAH + BSO + CT	Dead, 10
20	Ahmad et al. (2017)	48	No	ШB	Whitish vaginal discharge and lower abdominal pain	CT + RT	AWD, NA
21	Wang et al. (2017)	48	No	ⅡB	Irregular uterine bleeding	CT + RH + BSO + PL + CT	AWD, 27
22	Wang et al. (2017)	43	No	ⅡB	Urinary frequency	CT + RH + BSO + PL + CT	AWD, 12
23	Gupta et al. (2020)	20	No	ⅠB2	Foul smelling white discharge	TAH + PL + CT	AWD, 44
24	Kyriazoglou et al. (2019)	38	Yes	ⅠB2	Incidental finding	RH + BSO + PL + CT + RT	AWD, 42
25	Rajendra et al. (2020)	19	No	ⅣB	Vaginal bleeding, abdominal pain, general weakness, and anorexia	NO	Died
26	Feng et al. (2021)	19	Yes	ⅠB1	Vaginal bleeding and abdominal pain	RH + BSO + PL	AWD,36

PNETs, primitive neuroectodermal tumors; FIGO, International Federation of Gynecology and Obstetrics; AWD, alive without disease; DOD, died of disease; TAH, total abdominal hysterectomy; BSO, bilateral salpingo-oophorectomy; PL, pelvic lymphadenectomy; RH, radical hysterectomy; RT, pelvic radiotherapy; CT, chemotherapy; NA, not available; CRS, cryoreductive surgery.

The diagnosis of PNET mainly depends on the histopathological morphology, IHC examination, and molecular genetic analysis. In hematoxylin-eosin staining, the tumor is composed of small round cells of uniform size with indistinct cell boundaries and little cytoplasm. Homer-Wright rosettes, a characteristic manifestation of PNET, are seen in approximately 30–70% of cases (Tsao et al., 2001). The IHC markers currently used in the diagnosis of PNET include CD99 (also designated as MIC2 or HBA-71), neurofilament proteins, NSE, vimentin, FLI-1, and SYN (Patel et al., 2020). CD99 represents the most specific marker for the diagnosis of PNET, and in our case, it was intensely and diffusely expressed on tumor cell membranes. Occasionally, the expressions of neuroendocrine markers such as NSE, CgA, SYN, CD56, and S-100 are different, indicating neuroectoderm differentiation. The epithelial marker desmin might be focal positive. A characteristic chromosomal translocation t (11;22) (q24;q12), resulting in the *EWS-FLI1* fusion, was found in more than 90% cases, and other fusion genes include *ERG*, *ETV1*, *E1AF*, and *FEV* (Dedeurwaerdere et al., 2002; Teicher et al., 2011). Different combinations of chromosome breakpoints lead to strong heterogeneity at the molecular level. In general, breakpoints in *EWS* occur between introns 7–9, while breakpoints in *FLI1* occur between introns 3–9. Frequently, the 7th exon of *EWS* is fused to the 6th exon (60%) or to the 5th exon (20%) of *FLI1*, creating type 1 and type 2 *EWS-FLI1* fusions, respectively. Other variations of the *EWS-FLI1* fusion transcript have a lower incidence. Two independent retrospective studies showed the types of *EWS-FLI1* fusion associated with different clinical outcomes. Patients without metastatic disease usually had type 1 *EWS-FLI1* fusion, and higher event-free and overall survival rates than patients with type 2 fusion (De Alava et al., 2000; van Doorninck *et al.*, 2010). These studies suggest that the *EWS-FLI1* fusion type can be used to predict outcome at the time of diagnosis. The diagnostic criteria proposed by Schmidt et al. is the combination of tumor histomorphological characteristics, plus the positive immunohistochemical marker CD99 and the positive of two or more different neural markers (Schmidt et al., 1991). Boldorini et al. considered that FISH and RT-PCR detection of fusion genes are the gold standards, especially for the detection of PNET in rare sites (Boldorini et al., 2010). In our case, the IHC marker CD99 was diffusely positive, SYN and FLI-1 were partially expressed, and we confirmed the presence of the *EWS-FLI1* fusion gene by FISH. And RT-PCR results indicated that the patient displayed type 2 *EWS-FLI1* fusion, indicating a dismal prognosis and poor clinical outcome. Currently, positron emission tomography/computed tomography (PET/CT) after injection of Gadolinium-68-labeled somatostatin receptor binding Dota-octreotide (Dotanoc) is the preferred technique for functional imaging of neuroendocrine tumors, because the better histologically differentiated forms show an overexpression of somatostatin receptors at their cell surfaces. The specificity and sensitivity were 100 and 96%, respectively (Ambrosini et al., 2011). Gadolinium-68-Dotanoc PET/CT showed great advantages in the diagnosis and staging of neuroendocrine tumors. However, this method has not been widely carried out in China. In addition, PET/CT is radioactive, which limits its use in pregnant women. We performed chest computed tomography, abdominal and pelvic MRI instead to assess the staging of this patient. The results showed no lymph nodes or distant metastases.

Primary PNET of the female reproductive system is relatively rare in clinical cases, and is a highly aggressive malignant tumor with high mortality and short-term recurrence and metastasis. Because cervical PNET belongs to the family of Ewing’s sarcoma, the current treatment strategy is surgery combined with adjuvant chemotherapy. The role of radiation is unclear. More than 80% of patients who only underwent surgery would develop tumor relapse. The overall survival was significantly improved by combining surgery with chemotherapy (Snijders-Keilholz et al., 2005). Common chemotherapy drugs include cyclophosphamide, ifosfamide, vincristine, actinomycin-D, and doxorubicin. There is no standard chemotherapy regimen for PNET, and some recent case reports have shown longer disease-free intervals after treatment with platinum and etoposide (Thorn et al., 2016). Radiotherapy is usually used for patients with inoperable tumors and/or positive surgical margins, as well as for those with poor histological results (Wang et al., 2017). In the future, specific antibodies, such as antibodies of IGF-1, and cellular immunotherapy may be applied to inhibit PNET growth (Toretsky et al., 2001). In our case, after total hysterectomy and bilateral salpingo-oophorectomy and pelvic lymphadenectomy, we applied pirarubicin, ifosfamide, and cisplatinum chemotherapy every 3 weeks for six cycles and regional radiotherapy.

The overall prognosis of PNET is very poor, with a 5-year overall survival rate of less than 30%. Late stage, insufficient surgical resection and adverse effects of chemotherapy appear to be associated with unfavorable prognosis (Odunsi et al., 2004). At the time of writing, the patient had completed the treatment and experienced significant remission. Previously, two patients suffering from cervical PNET were treated with the same chemotherapy regiment in our department, and they remained free of disease for sixty and seventy-five months, respectively.

## Conclusion

PNET in the female reproductive tract is an extremely rare disease. In this article, we reported a case of cervical PNET complicated by pregnancy. The diagnosis of PNET depends on pathology, immunohistochemistry, and genetic analysis. Molecular analysis may significantly contribute to the final diagnosis of PNET occurring in this unusual location. In addition, the type of *EWS-FLI1* fusion may be related to clinical outcomes. At present, the effective treatment for PNET is surgery combined with chemotherapy and radiotherapy. The rapid growth of PNET during pregnancy may be related to hormonal changes and further research is needed.

## Data Availability

The datasets for this article are not publicly available due to concerns regarding participant/patient anonymity. Requests to access the datasets should be directed to the corresponding author.
